# Myocyte Remodeling Due to Fibro-Fatty Infiltrations Influences Arrhythmogenicity

**DOI:** 10.3389/fphys.2018.01381

**Published:** 2018-10-04

**Authors:** Tim De Coster, Piet Claus, Gunnar Seemann, Rik Willems, Karin R. Sipido, Alexander V. Panfilov

**Affiliations:** ^1^Department of Physics and Astronomy, Ghent University, Ghent, Belgium; ^2^Department of Cardiovascular Sciences, KU Leuven, Leuven, Belgium; ^3^Institute for Experimental Cardiovascular Medicine, University Heart Centre Freiburg • Bad Krozingen, Medical Center – University of Freiburg, and Faculty of Medicine, University of Freiburg, Freiburg, Germany; ^4^Laboratory of Experimental Cardiology, Department of Cardiology, Heart Lung Centre Leiden, Leiden University Medical Center, Leiden, Netherlands; ^5^Laboratory of Computational Biology and Medicine, Ural Federal University, Ekaterinburg, Russia

**Keywords:** atrial fibrillation, adipose tissue, ionic modeling, arrhythmogenicity, computational modeling

## Abstract

The onset of cardiac arrhythmias depends on the electrophysiological and structural properties of cardiac tissue. Electrophysiological remodeling of myocytes due to the presence of adipocytes constitutes a possibly important pathway in the pathogenesis of atrial fibrillation. In this paper we perform an *in-silico* study of the effect of such myocyte remodeling on the onset of atrial arrhythmias and study the dynamics of arrhythmia sources—spiral waves. We use the Courtemanche model for atrial myocytes and modify their electrophysiological properties based on published cellular electrophysiological measurements in myocytes co-cultered with adipocytes (a 69–87 % increase in *APD*_90_ and an increase of the RMP by 2.5–5.5 mV). In a generic 2D setup we show that adipose tissue remodeling substantially affects the spiral wave dynamics resulting in complex arrhythmia and such arrhythmia can be initiated under high frequency pacing if the size of the remodeled tissue is sufficiently large. These results are confirmed in simulations with an anatomically accurate model of the human atria.

## 1. Introduction

Atrial fibrillation (AF) forms an economic burden on modern health care (Stewart et al., 2004). It affects approximately 1.5 % of the population (Lip et al., 2007) and as much as 9% of people above the age of 80 (Go et al., 2001). AF is a progressive disease and results in increased mortality, morbidity, and impaired quality of life (Thrall et al., 2006). AF is therefore a major problem in cardiac electrophysiology and obtaining insights into the mechanism of this arrhythmia is of great interest to progress therapeutic strategies.

Several risk factors for AF have been identified. Of interest here, obesity increases the risk of AF by 49% in the general population, and the risk rises with an increased body mass index (Wanahita et al., 2008). It was shown that it is an independent risk factor for the occurrence of AF or progression of AF severity in the absence of other risk factors like heart failure, alcohol use, or hypertension (Wang et al., 2004; Dublin et al., 2006; Tsang et al., 2008). Recent experiments have shown significant correlations between adipose infiltrations and arrhythmias (Pantanowitz, 2001; Samanta et al., 2016; Haemers et al., 2017).

Risk factors result in physiological stimuli for the atrial myocardium resulting in a substrate for AF. Traditionally, structural factors like fibrosis, but also heterogeneities in atrial action potential have been identified. However, adipose tissue can affect the onset of arrhythmias as it produces both structural and electrophysiological (Lin et al., 2010) changes.

Recently a new detailed study examined the relationship between fatty infiltrations in the atrial myocardium and the development of AF substrate (Haemers et al., 2017). There, a direct relation between fibro-fatty infiltrations and arrhythmia was shown. Induction of AF in a sheep model resulted in a fibrotic remodeling of subepicardial adipose tissue infiltrates from pure fatty to dense fibro-fatty infiltrations and smaller adipocytes in the atrial wall. This remodeling was associated with cellular inflammation.

Adipose tissue creates in-excitable regions inside the heart similar to fibrosis (Pantanowitz, 2001). Structural dependency of arrhythmogenicity on adipocytes was recently studied *in-silico* in a generic two-dimensional model (De Coster et al., 2018). It was shown that a substrate with adipocytes is less arrhythmogenic than with fibrotic tissue and thus during remodeling of fatty into fibro-infiltrations the propensity to arrhythmias increases, confirming the results of Haemers et al. (2017).

In addition to creation of in-excitable regions inside the heart, adipose tissue also causes electrophysiological changes in the adjacent myocytes. Co-culture of rabbit atrial myocytes and epicardial adipocytes, results in remodeling of the myocyte action potential (Lin et al., 2012). The consequences at organ level of this remodeling have not yet been studied. However, they may substantially contribute to arrhythmogenesis in addition to structural effects. Furthermore, these changes can be more abundant as electrophysiological changes do not require infiltration of fat into cardiac tissue and occur even due to the presence of epicardial fat.

In this paper, we describe changes in properties of myocytes adjacent to adipocytes on the basis of an experimental study (Lin et al., 2012). The aim of this study was to investigate the effect of this adipose remodeling on the wave dynamics of spirals, onset of arrhythmias, and influence on its surroundings. We performed use *in-silico* simulations in a generic two dimensional setup and in a realistic anatomical model of human atria.

## 2. Methods

### 2.1. Mathematical model

To study the effect of remodeled myocytes in the vicinity of adipocytes in human cardiac tissue, we integrated a newly developed single cell model (explained in the next subsection: myocyte remodeling) together with the Courtemanche model (Courtemanche et al., 1998, 1999) for normal human atrial myocytes. The transmembrane voltage (*V*) is calculated in millivolts (mV) according to the mono-domain equations for cardiac tissue (Equation 1):

(1) $$\frac{\partial V}{\partial t}=\nabla \left(D\nabla V\right)-\frac{I_{ion}}{C_{m}},$$

where *t* is time in milliseconds (ms), *I*_*ion*_ is the total ionic current density in microamperes per square centimeter (μA/cm^2^) and is dependent on *V*, on the gating variables, and on the concentrations of intracellular calcium, *C*_*m*_ is the specific membrane capacitance in microfarad per square centimeters (μF/cm^2^), and **D** is the diffusion tensor, whose components are related to the myocyte orientation and gap junction distribution which create anisotropy.

A maximum velocity planar wave propagation of 54.71 cm/s at a stimulation frequency of 1 Hz was obtained for regular tissue.

For the human atrial morphology, data and fiber directions were obtained from Dössel et al. (2012). The ionic cell models that were used are the regular and the AF-induced electrically remodeled human atrial Courtemanche model (Courtemanche et al., 1998, 1999) (taken from CellML Yu et al., 2011 with the use of Myokit Clerx et al., 2016 and transformed to GPU-usable code).

We used an S1-S2 cross-field protocol to generate spiral and scroll waves. In human atrial monolayers, we assumed isotropy. Thus, the diffusion tensor of Equation (1) could be replaced by a scalar coefficient *D*. However, in 3D, to account for the anisotropy of realistic cardiac tissue, the diffusion tensor components *D*_*ij*_ were scaled according to the fiber direction tensor. The transverse diffusion coefficient (*D*_*t*_, for signal propagation across the fibers) was assumed to be 9 times less than the longitudinal diffusion coefficient (*D*_*l*_, for signal propagation along the fibers) giving rise to a realistic conduction velocity ratio (Liu et al., 2004; Aslanidi et al., 2011). Explicitly,

(2) $$\begin{array}{r} D_{ij}=\left(D_{l}-D_{t}\right)\alpha_{i}\alpha_{j}+D_{t}\delta_{ij}, \end{array}$$

where α_*i*_ are components of the unit vector that is oriented along the direction of a fiber and *D*_*l*_ = 1.3 cm^2^/s.

### 2.2. Myocyte remodeling

We used the Courtemanche model for both the normal (Courtemanche et al., 1998) and AF remodeled (Courtemanche et al., 1999) human atrial myocyte. The total ionic current consists of the following currents:

$$\begin{array}{rc} I_{ion} & =I_{Na}+I_{CaL}+I_{K1}+I_{to}+I_{NaCa}+I_{NaK}+I_{Kur}+I_{Kr}+I_{Ks} \end{array}$$

(3) $$\begin{array}{r} \;+I_{bNa}+I_{bCa}+I_{pCa}. \end{array}$$

Here *I*_*Na*_ represents the fast *Na*^+^ current, *I*_*CaL*_, the L-type *Ca*^2+^ current, *I*_*K*1_, the inward-rectifier *K*^+^ current, *I*_*to*_, the transient outward *K*^+^ current, *I*_*NaCa*_, the *Na*^+^/*Ca*^2+^ exchanger current, *I*_*NaK*_, the *Na*^+^/*K*^+^ pump current, *I*_*Kur*_, the ultra-rapid-delayed rectifier *K*^+^ current, *I*_*Kr*_, the rapid-delayed rectifier *K*^+^ current, *I*_*Ks*_, the slow-delayed rectifier *K*^+^ current, *I*_*bNa*_, the background *Na*^+^ current, *I*_*bCa*_, the background *Ca*^2+^ current, and *I*_*pCa*_, the plateau *Ca*^2+^ current.

The AF remodeled atrial cell model has changes in 3 of these ionic currents, namely a 70% reduction in *I*_*CaL*_, a 50% reduction in *I*_*to*_ and a 50% reduction in *I*_*Kur*_, resulting in a decreased action potential duration (APD).

**Figure 1:**
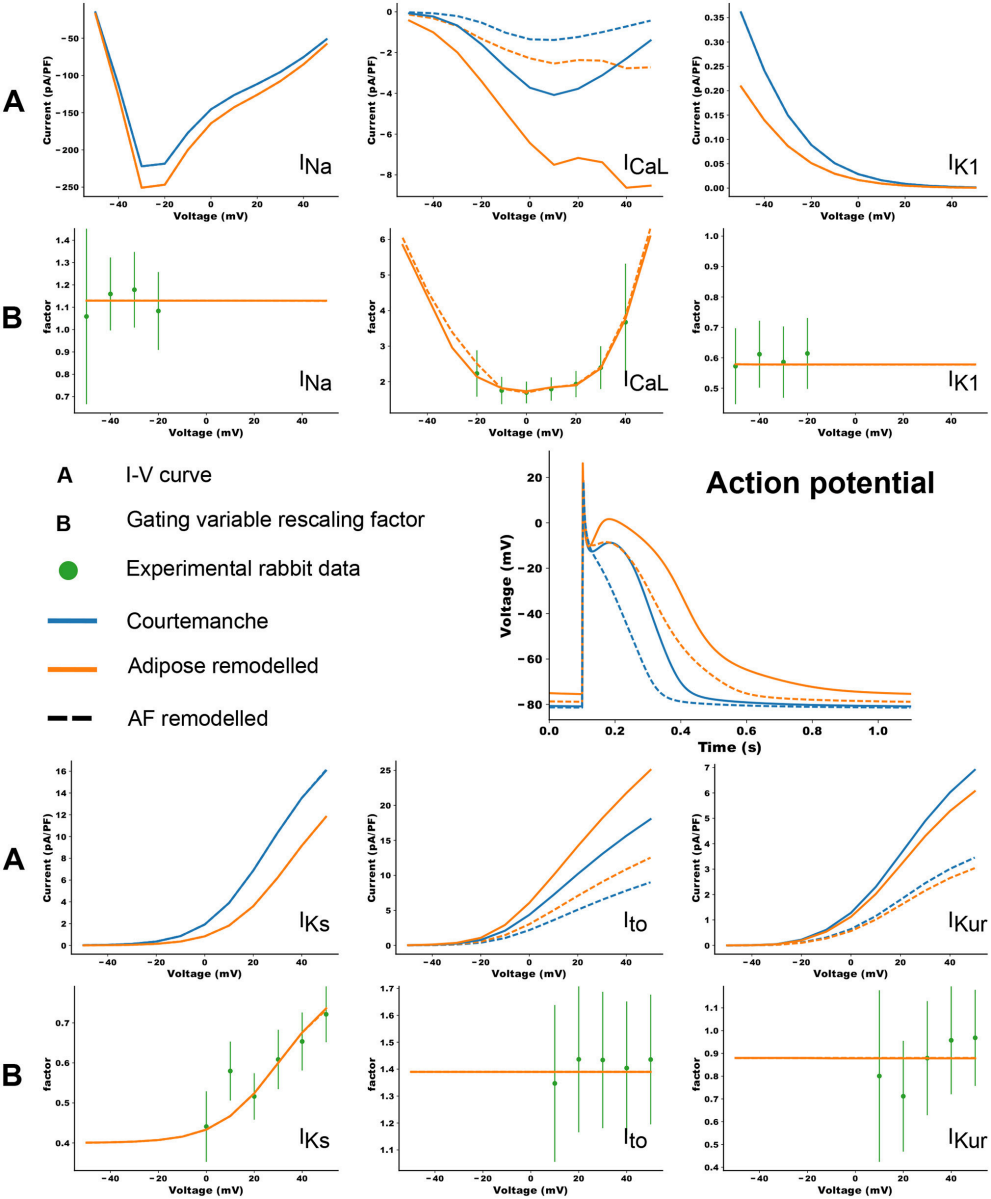
The changes in ionic currents due to the presence of adipose tissue. The peak values of the currents are presented. The blue curves denote the IV curves and action potential of the Courtemanche model, while the orange curve denotes the same with additional changes due to remodeling induced by the adipose tissue. Dashed curves show the data for additional AF remodeling. The experimental measurements and error bars are extracted from Lin et al. (2012) and shown in green. In orange the *in silico* result based on the factors in Table 1 is shown. More information can be found in the text.

Co-culture of adipocytes and left atrial myocytes has revealed important changes in ionic currents in a rabbit atrial model (Lin et al., 2012). Measurements were made for co-culture of myocytes with adipose tissue from different regions in the heart (epicardial, retrosternal), abdominal adipose tissue, and adipocytes-conditioned supernatant for 2–4 h. We focused on remodeling due to epicardial adipose tissue as we aim to look at the arrhythmogenic effect of infiltrates. Based on these experimental data, we adapted 6 ion currents for adipose induced myocyte remodeling, namely *I*_*CaL*_, *I*_*to*_, *I*_*Kur*_, *I*_*Na*_, *I*_*Ks*_, and *I*_*K*1_. In Lin et al. (2012) the tail component of *I*_*Kr*_ was measured. Its dynamics resembles strongly *I*_*Ks*_ and it was therefore chosen as a replacement since the Courtemanche model does not have a tail component of *I*_*Kr*_ current. Experimental data points and corresponding error bars were extracted for the I-V plots in Lin et al. (2012). In Figure 1B, we present a *relative* I-V plot for the six relevant ion currents in remodeled myocytes. To obtain these plots, the remodeled peak currents were divided by the peak current of the normal myocyte. The obtained data points together with their corresponding error bar are denoted in green. These ratio's are assumed to be species independent and are consequently used to validate ionic changes made to a human atrial myocyte model. To inflict these changes, a combination of functions was tested to rescale the gating variables of the selected currents. These functions were either linear, parabolic or a sigmoid (see Table 1). After rescaling, simulations were run in a single cell, mimicking patch clamp experiments, to construct a current-voltage (I-V) relationship from *in silico* data. Model curves (Figure 1A) represent peak currents measured during 3,000 ms voltage steps to various potentials from a holding potential of –80 mV (This method was validated against earlier results, see Figure S1 in the Supplementary Information). The peak values of adipose remodeled and regular cells were divided by each other to obtain a factor that can be compared with the experimental one. These data were presented by means of a full or orange dotted line on top of the experimental data (see rows denoted by the letter B in Figure 1). It can be seen that all executed conduction factor changes make for results that are in correspondence with experiment.

**Table 1:** Correction factors for selected currents in the human atrial model, based on measurements and fits to rabbit atrial data (rows denoted by the letter B in Figure 1).

	**Best fit**	**Value**
$g^{\prime }{\left(V\right)}_{Na}$	Constant	1.13
	Parabolic	*V* < 0*mV*: 0.0017*V*^2^+1.955
$g^{\prime }{\left(V\right)}_{CaL}$	Sigmoid	0*mV*<*V* < 20*mV*: $2.071+\frac{1.949-2.071}{1+e^{0.4\times \left(V-7.82mV\right)}}$
	Parabolic	*V*>20*mV*: 0.005 × (*V*−20.0)^2^+2.07
$g^{\prime }{\left(V\right)}_{to}$	Constant	1.39
$g^{\prime }{\left(V\right)}_{Kur}$	Constant	0.880
$g^{\prime }{\left(V\right)}_{Ks}$	Sigmoid	$0.8+\frac{0.4-0.8}{1+e^{0.08\times \left(V-30mV\right)}}$
$g^{\prime }{\left(V\right)}_{K1}$	Sigmoid	$0.578+\frac{0.805-0.578}{1+e^{0.3\times \left(V+70mV\right)}}$

The new maximal conductivity factor for the sodium current I_Na_ would now be $g^{\prime }{\left(V\right)}_{NA}*g_{Na}$.

The final results of the rescaling factors can be found in Table 1 and are used to scale the conductances. E.g. the new maximal conductance for the sodium current *I*_*Na*_ would now be $g^{\prime }{\left(V\right)}_{Na}*g_{Na}$, where $g^{\prime }{\left(V\right)}_{Na}$ is the measured curve, and *g*_*Na*_ is the standard maximal conductance. A table with all maximal conductances for all four models can be found in Table S1 in the Supplementary Information.

### 2.3. Pacing protocols

For all single cell simulations, we paced at a frequency of 1 Hz and considered the 12th action potential in agreement with previously published APs.

For 1D simulations, we obtained a restitution curve by means of a dynamic pacing protocol. The tissue was paced from one side at a basic cycle length (BCL) of 1,100 ms for six pulses. The BCL was progressively lowered with a step equal to 1 percent of the current BCL. This was done up until a step size of 1 ms was reached, after which we always went down with this minimal step size of 1 ms. This was repeated until conduction became impossible.

In the same cable configuration we obtained measurements of the conduction velocity from the twelfth pulse.

A variety of pacing protocols was tested in tissue monolayers. Firstly, we induced spirals by means of an S1S2-protocol. The first pulse was from one side of the tissue, while the second one comprised one eighth of the tissue just behind the waveback, going from the top of the tissue until halfway in the y-direction and from one quarter until the middle in the x-direction.

Secondly, to determine the minimal size of a remodeled patch to induce arrhythmia, we used an S1S2 protocol where the first pulse was from one side of the tissue, while the second pulse was delivered by a point located at the edge of the remodeled tissue patch.

Thirdly, a burst-pacing protocol for spontaneous spiral induction that consisted of 10 pulses at different BCL (1,000, 900, 800, 700, 600, 500, 450, 400, 350, 320, 300, 290, 280, 270, 260, 250, 240, 230, 220, all in ms) was used.

For anatomically correct human atrial data we used an induced spiral protocol. The spiral was induced at four locations, twice at a location in the left atrium and twice at a location in the right atrium and obtained by means of an S1S2-protocol. The first pulse was initiated from one side of the atria (the left atrium was chosen), while the second pulse is a slice through the whole atrium right after the waveback and perpendicular to the first pulse.

### 2.4. Implementation

We integrated the system of coupled ordinary differential equations Equation 1 in time using the forward Euler method with time step δ*t* = 0.005 ms, and in space, using the centered finite-differencing scheme with space step δ*x* = δ*y* = 0.0300 cm in monolayers, and also 0.0300 cm in whole atria, subject to “no flux” boundary conditions. Our monolayer domains contained 1024 × 1024 grid points, so that the physical size of the simulated tissue was 30.72 × 30.72 cm to reduce possible boundary effects. The simulation domain for the whole atria contained 2, 173, 891 grid points. The gating variables in the electrophysiological model for the human cardiomyocyte were integrated using the Rush and Larsen scheme (Rush and Larsen, 1978).

The numerical solver was implemented with the C and C++ programming languages, using the CUDA toolkit for performing the majority of computations on graphical processing units. Visualization of results was done with the help of the Python programming language and ParaView (Kitware). Computations were performed with single precision and run on an Intel Core i7-3930K machine with two GeForce GTX TITAN Black graphics cards.

## 3. Results

### 3.1. Single cell

Differences in the ionic currents of four models (regular, regular + AF remodeling, adipose remodeling, adipose remodeling + AF remodeling) resulted in differences in action potential shapes. Figure 1 shows these changes for 1 Hz pacing.

The results are shown in the rows denoted by the letter A in Figure 1. Epicardial adipocyte-incubated myocytes have larger late sodium currents (*I*_*Na*_), L-type calcium currents (*I*_*CaL*_) with an extra increase around its maximal value, and transient outward potassium currents (*I*_*to*_) than control myocytes. On the other hand we see smaller delayed rectifier potassium (*I*_*Kr*_ and *I*_*Ks*_) and inward rectifier potassium currents (*I*_*K*1_). The extent of change of these currents due to remodeling corresponds to the experimental values.

These changes in ionic currents result in the following changes in the cellular action potential. In adipose-remodeled myocytes, the resting membrane potential (RMP) increases, making depolarization of the cell easier. The action potential amplitude (APA) remains similar resulting in a higher maximal voltage reached. An increased calcium current results in a higher plateau-level and prolongation of the *APD*_50_. The decreased potassium rectifiers cause elongation of the tail (phase 3) of the remodeled AP, resulting in noticeable elongation of *APD*_90_. Numerical values for all aforementioned properties (RMP, APA, and APD) can be found in Table 2.

**Table 2:** Action potential properties for four models used in simulations.

	**Courtemanche**	**Adipose**	**AF**	**AF + adipose**
		**remodeled**	**remodeled**	**remodeled**
*APD*_90_ (ms)	299.910	507.545	215.425	403.330
*APD*_50_ (ms)	180.725	272.970	86.475	185.930
*APD*_20_ (ms)	5.680	4.635	7.660	6.235
*RMP* (mV)	−80.857	−75.466	−81.370	−78.841
*APA* (mV)	105.651	103.523	106.251	107.565

*Adipose remodeling makes the RMP higher, elongates the tail of the APD (The difference in APD_90_ is much larger than the other APD-measurements), and increases the action potential amplitude (APA) of the APD*.

Separate contributions of each current to the changes in action potential shape were also examined and can be found in the Supplementary Information: Figure S2.

### 3.2. 1D propagation

#### 3.2.1. Conduction velocity

We calculated the conduction velocity in a 1D cable configuration. For a 1 Hz stimulation frequency the conduction velocities of the four different models with the standard value of the diffusion coefficient of 1.3 cm^2^/s are: 50.94 cm/s for the Courtemanche model, 50.96 cm/s for the AF remodeled Courtemanche model, 56.04 cm/s for the adipose remodeled atrial myocyte, and 54.88 cm/s for the AF and adipose remodeled atrial myocyte. These results are consistent and correspond to conduction velocities observed in experiment (AF that was characterized by one or two non-uniformly conducting wavelets corresponds to 54±4 cm/s; Konings et al., 1994).

#### 3.2.2. Restitution curve

**Figure 2:**
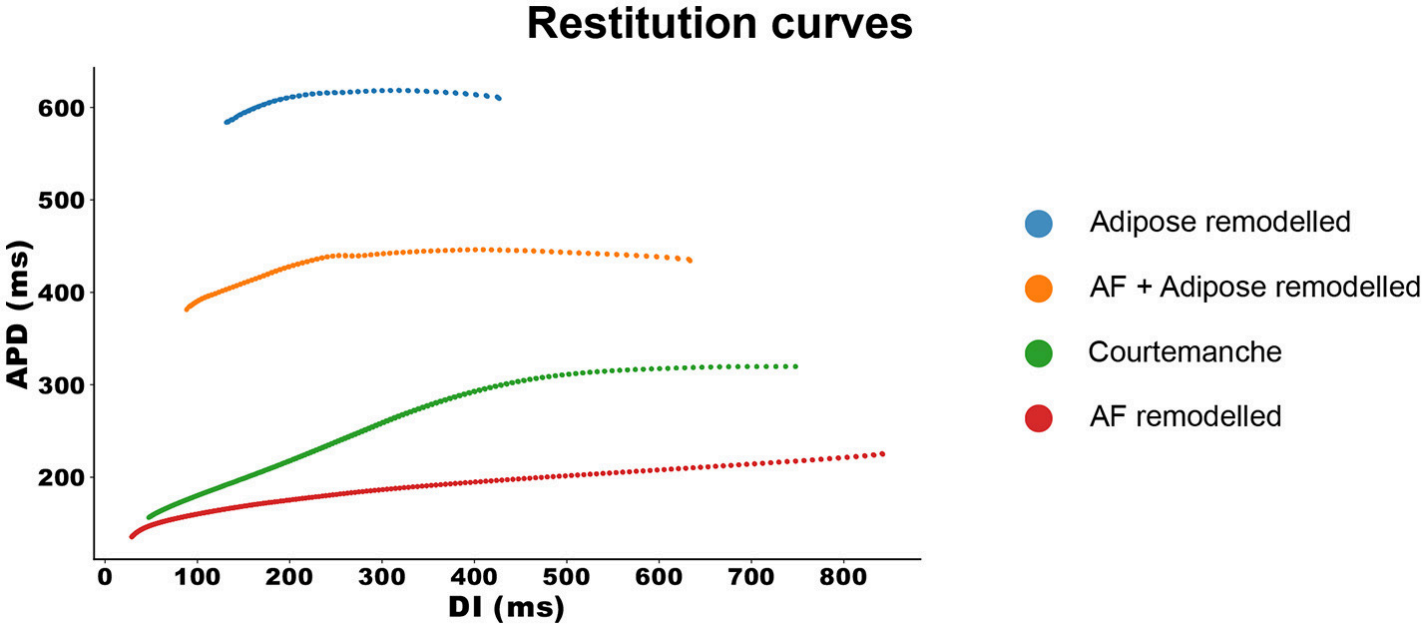
Restitution curves for all four models. The APD measurements are made at 90% repolarization.

With the same configuration, we investigated the APD restitution properties of all four models. The results can be seen in Figure 2.

The Courtemanche model, depicted in green, has a regular restitution curve with a decrease in APD for low diastolic intervals (DIs). The AF remodeled Courtemanche model (the red line) shows a slow decrease in APD which becomes more steep for lower DI (see also Supplementary Information Figure S3). When adipose remodeling is considered, restitution curves change. Adipose tissue remodeling of the regular Courtemanche model (the blue line), results in substantial prolongation of APDs and a decrease happens at lower DIs with a slope similar to that of the Courtemanche model. Adipose remodeling of the AF Courtemanche model (the orange line) results in an almost flat dependency for long DIs and slow decrease at small DIs. There is also substantial difference in maximal pacing rate between the models. For the normal Courtemanche model the maximal frequency of pacing was 4.9 Hz, for the AF remodeled Courtemanche model it is 6.1 Hz, however for the adipose remodeled tissue it was 1.4 Hz and for the AF remodeled adipose tissue it was 2.1 Hz.

### 3.3. Tissue monolayers

Next we study how observed changes in electrophysiological properties will affect 2D dynamics of the arrhythythmias generated by spiral waves for the four different models as well as for combinations of the models in case of heterogeneous cardiac tissue.

#### 3.3.1. Properties of spiral waves in normal and remodeled tissue

**Figure 3:**
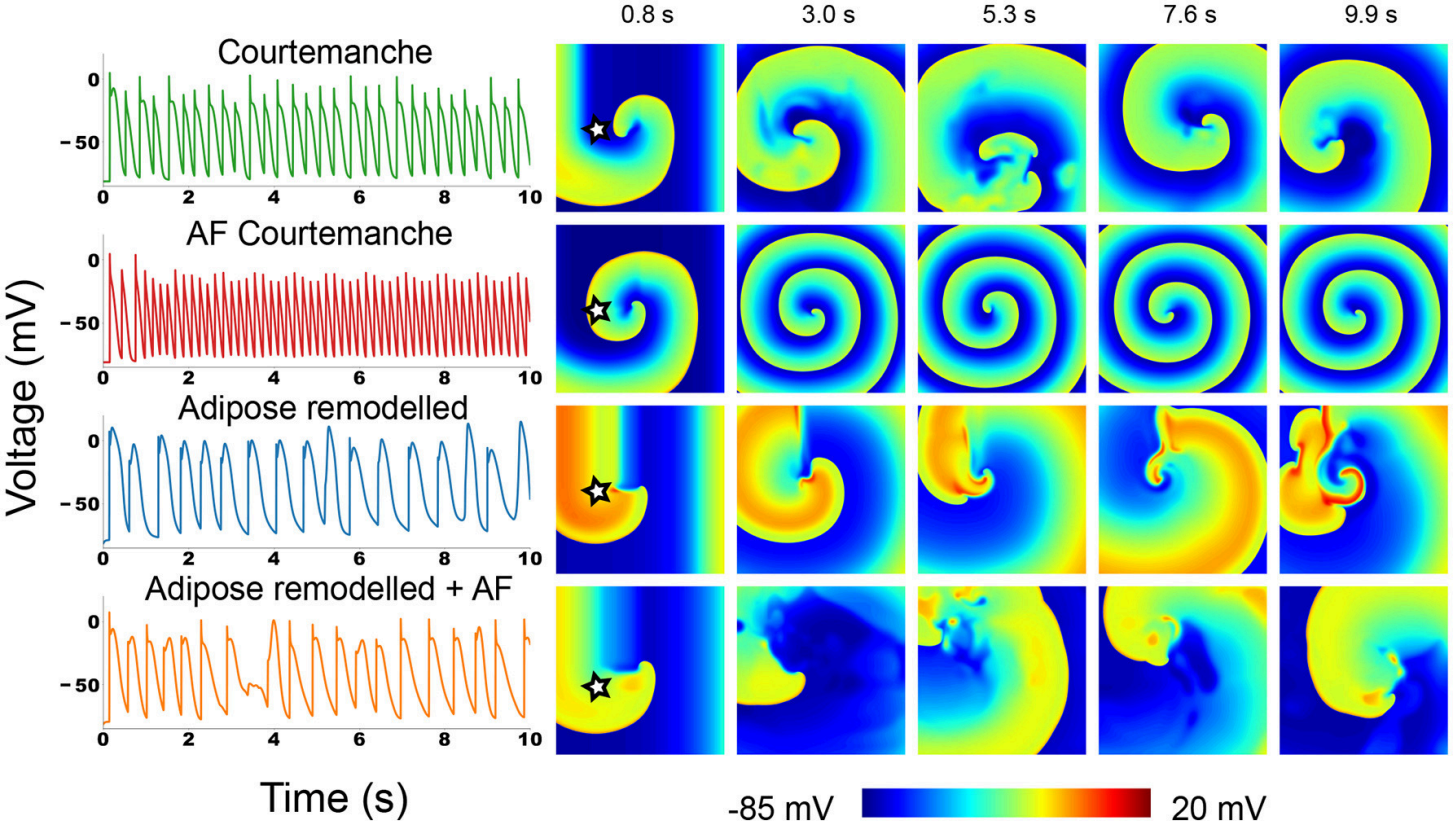
Action potential traces and voltage snapshots of an S1S2 induced spiral in all four of the studied atrial myocyte models. The action potential traces were taken at the point denoted by a star in the first depicted time frame.

We induced a spiral wave by an S1-S2 protocol in 2D homogeneous tissue for each of the four tissue types and studied its dynamics. The representative snapshots and the voltage recordings at one selected point are presented in Figure 3. Full movies can be found in the Supplementary Information.

In the Courtemanche model (top row in Figure 3) we can see a meandering spiral. The action potential trace shows small variation of atrial APs in time due to the meandering, but the changes are small. This is in line with previous observations in the Courtemanche model (Courtemanche et al., 1998). The AF remodeled Courtemanche model (second row in Figure 3) also shows a stable spiral with almost completely periodic action potentials. Compared to the normal Courtemanche model, the core of the spiral and the wavelength becomes much smaller. These dynamics also correspond to what has previously been observed for this model (Courtemanche et al., 1999).

For adipose remodeled tissue in both cases we observe completely different dynamics: the complexity of the pattern increases due to multiple successive break-ups of the spiral.

The regular Courtemanche model with adipose remodeling (third row in Figure 3) shows initially regular spiral behavior. However, temporary block in the rotating spiral makes for interesting changes in the behavior of the cells. The characteristic feature of the observed pattern are the high voltage plateaus (see Supplementary Information Video S1). They create block that initializes the first spots where reentry can occur. However, due to the elongated tail of the action potential when a wave passes this region, its propagation is further disturbed which again can mediate block. In this way an irregular pattern is slowly generated that remains present. The spatio-temporal irregularity increases in time.

In the model where both AF remodeling and adipose remodeling were applied (bottom row in Figure 3), we see similar behavior. The initial spiral undergoes multiple break-ups in the course of time. We can see that this is due to calcium dynamics. The break-up is however less pronounced than in the previous case. The disturbance is lower than in the previous case, but is still able to produce temporal block. On top of this phenomenon, we observe that the spiral rotation period decreases due to the AF remodeling. This makes that after such a temporal block, a quick rotation follows. This makes the curvature of the wavefront become too large and results in additional breaks. Once again this results in complex spatio-temporal excitation patterns.

Overall we can conclude that adding the adipose remodeling results in larger complexity of the spiral wave dynamics.

#### 3.3.2. Effect of an adipose remodeled patch on spiral wave dynamics

In normal conditions the epicardial fat occupies only a part of the atrial tissue. We therefore also studied how the presence of a patch of a certain size of adipose remodeled tissue will affect the dynamics of an existing spiral wave. We investigated circular adipose remodeled patches placed into normal or AF remodeled tissue. We induced arrhythmia by an S1S2 mechanism.

**Figure 4:**
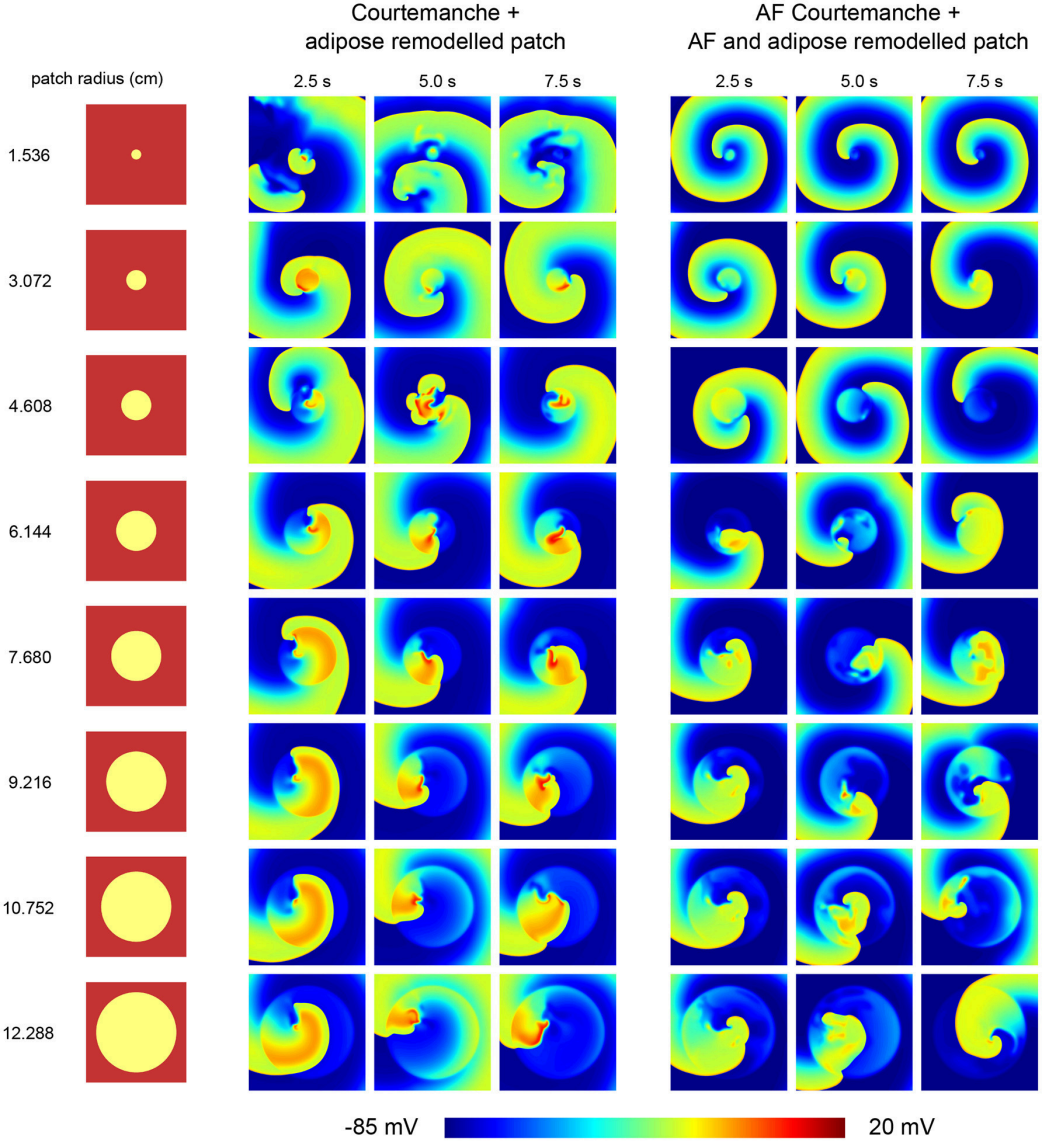
Effect of an adipose remodeled patch on spiral wave dynamics. Different tissue configurations are shown on the left, indicating the size of the remodeled patch. The resulting spiral wave dynamics upon S1S2 spiral initiation are shown to the right of the corresponding tissue configuration. Two cases were considered. One with the Courtemanche model and an adipose remodeled patch. Another one with the AF Courtemanche model and an AF and adipose remodeled patch. Three regimes are observed for the spiral wave dynamics: spiral wave anchoring, spiral wave spatio-temporal irregularity, and a spiral influencing remodeled patch. For more details regarding these regimes, see the main text.

Depending on the size of the arrhythmia and the combination of tissue, different cases of wave dynamics are observed (Figure 4).

The first case consists of spiral wave anchoring. Upon initiation of the spiral, it quickly attaches itself to the boundary of the adipose remodeled patch, after which it rotates around and, due to its longer APD duration periodically invades the patch itself. This is the case for the two smallest radii (1.536 and 3.072 cm) when having the normal Courtmanche model with an adipose remodeled patch. In case of the AF Courtemanche model with an AF and adipose remodeled patch, this behavior occurs for larger radii as well (up to 6.144 cm). In both cases, the spiral takes longer to anchor itself the larger the radius of the patch is in case it gets initiated in the center of the patch. For the smallest radius of the regular Courtemanche model with adipose remodeled patch, this was difficult to accomplish and it can be seen that it takes a while before this happens (see Video S2).

The second case consists of spiral wave spatio-temporal irregularity. When the spiral is initiated in the middle of the patch, it creates wave-blocks by means of the methods described earlier. This process stays inside the patch and creates irregular activation patterns. The radii where this behavior occurs are the five largest ones for the leftmost tissue configuration presented in Figure 4 (6.144, 7.680, 9.216, 10.752, and 12.288 cm), and the four largest ones for the rightmost configuration.

And finally, the third case consists of a spiral influencing remodeled patch. It was observed in one instance: a radius of 4.608 cm with a Courtemanche and adipose remodeled patch configuration. Inside the patch some irregular behavior is present. Over the course of time, this irregular behavior produces waves which it pushes outside of the patch which can be seen in Figure 4 in the 5 s frame. These irregularities influence the spiral wave behavior, making that the spiral doesn't attach to the patch but rather moves in close proximity of it. This case can be seen in the Supplementary Information in Video S3.

Upon inducing a spiral inside a remodeled patch, the arrhythmia is sustained but varies in complexity depending on the radius of the patch.

#### 3.3.3. Effect of an adipose remodeled patch on spiral wave initiation

We have also studied the possibility to induce arrhythmia by high frequency pacing due to the presence of an adipose remodeled patch.

In the previous section a spiral was induced *ad-hoc*. We also looked at spontaneous induction of spirals. We did this by means of an S1S2 protocol that could initialize a figure of 8 reentry pattern. However, when trying this protocol on both cases (regular and an adipose remodeled patch, and AF remodeled and an AF plus adipose remodeled patch), it was observed that patches are needed that have an unphysical dimension (radius larger than 11 cm) to induce this kind of reentry. Therefore, in real atria, arrhythmia will not spontaneously induce itself at low frequencies as the patch sizes that can fit in an atrium are smaller.

Since the size of a patch is a limit to its arrhythmogenicity, we looked to the minimal frequency to get arrhythmia in a patch as well. The size we used to measure this minimal frequency is a radius of 4.608 cm. This radius was chosen for both cases, regular and adipose remodeled patch, and AF remodeled and AF plus adipose remodeled patch. This particular choice was made since that radius constitutes the regime where you switch between an attached spiral and spiral wave spatio-temporal irregularity. We found that in the first case, with regular tissue and an adipose remodeled patch, the first spontaneous break-up occurs between a frequency of 1.3 and 1.4 Hz or otherwise said a BCL shorter than 800 ms, but larger than 700 ms. When we look at AF remodeled tissue with an AF plus adipose remodeled patch, we observe a minimal frequency between 2.0 and 2.2 Hz or a BCL that has to be smaller than 500 ms but larger than 450 ms.

### 3.4. Realistic human atria

**Figure 5:**
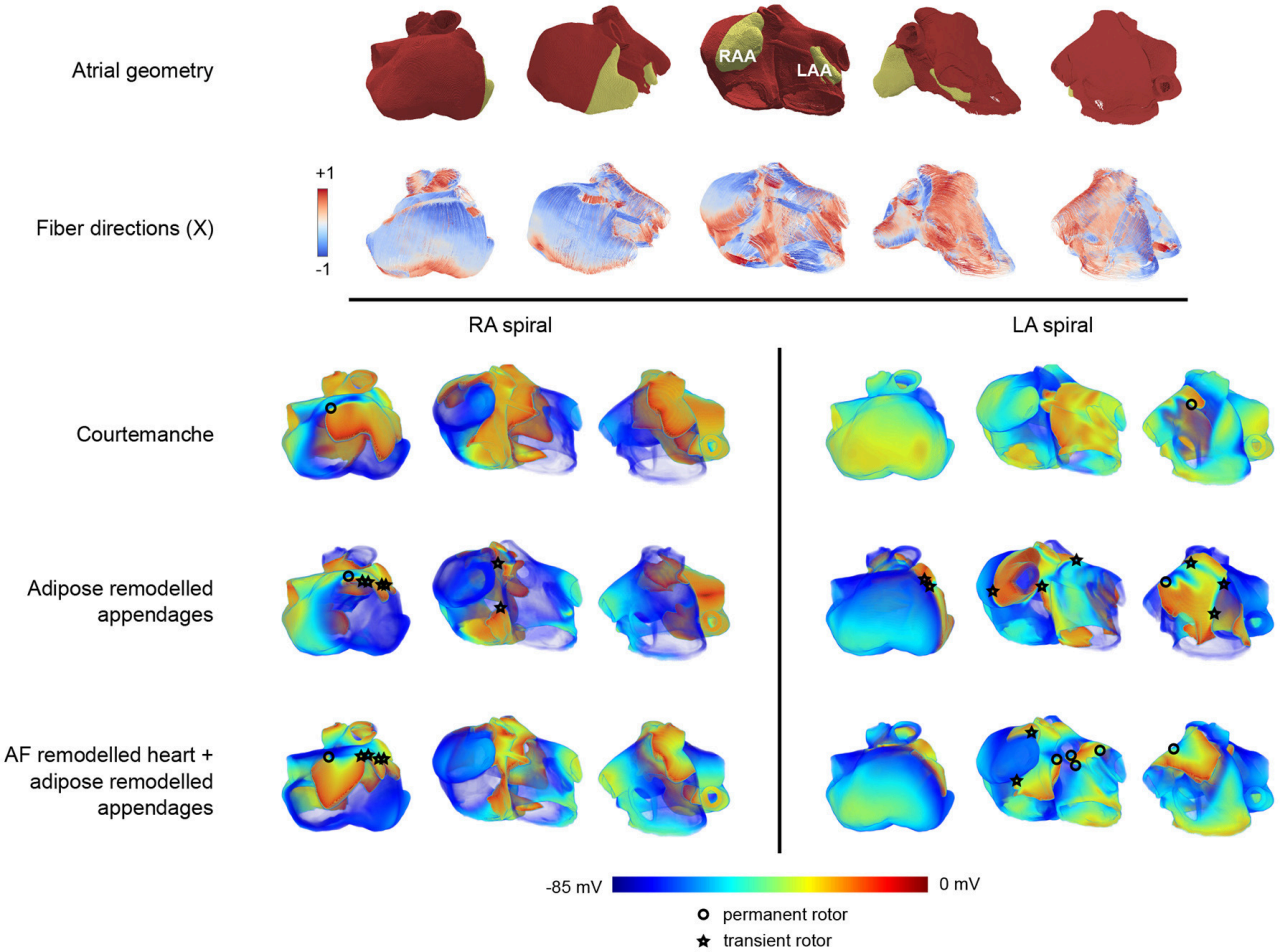
Arrhythmia patterns in three dimensional realistic atria.The top two rows of panels show the configuration in five different orientations. The top row provides a view on the geometry of the atria where the appendages are colored in yellow, and the remaining part of the atria is colored in red. The second row provides the corresponding fiber orientation colored according to its orientation along one of the axes (X). In these five panels, the leftmost orientation provides a view on the right atrium, while the rightmost one provides a view on the left atrium. The panels in between show a rotation between those two configurations showing the front view of the atria. In the voltage maps at the bottom only the leftmost, middle, and rightmost configuration are presented. The three lower rows (split into two columns) show one frame out of an S1-S2 induced arrhythmia in three different orientations. Each row corresponds to the tissue types that were used (Courtemanche, Courtemanche + adipose remodeled appendages, AF Courtemanche + AF and adipose remodeled appendages). Each column corresponds to the location where the arrhythmia was initialized (in the right atrium, or in the left atrium). One can see different kinds of electrical wave propagation complexity for the different combinations present. The cores of spirals are denoted by either circles or stars. The circle denotes a rotor that remains during the whole arrhythmia simulation. The star indicates that the rotor disappears over the course of time.

After studying effects of adipose remodeling in two dimensions, we performed similar studies in an anatomical model of human atria. Clinical and experimental observations show that epicardial fat occurs at the left or right atrial appendages. Figure 5 shows the anatomy of the human atria in our model and also highlights the appendages and the fiber directions.

#### 3.4.1. S1S2 spiral induction

We have first studied dynamics of spiral waves induced by an S1-S2 protocol for three configurations. The first one consisted of the homogeneous atrium with the Courtemanche model. The second one was the same but with both atrial appendages adipose remodeled. The third configuration consisted of the whole tissue being AF remodeled and the atrial appendages being AF and adipose remodeled. Spirals were induced outside of the atrial appendages to see what the effect of the remodeled appendages would be on the dynamics of the arrhythmia. We considered two placements of the spiral wave: in the left atrium and in the right atrium. Each case was simulated twice: two locations for the spiral were chosen in the right atrium, and two in the left atrium. Results were similar for different locations of the spiral in the left or right atrium. The results of one of the locations in each half of the atria can be seen in Figure 5. One representative frame out of each simulation is shown.

#### 3.4.2. Spiral wave dynamics

Upon initiation of the spiral wave for the first case (regular Courtemanche model everywhere), we are able to see that the spiral remains in the atria at all times and does not create additional complexity or break-up. Some distortions of the wave-front are observed however, though they do not change the overall dynamics of the arrhythmia, neither in the left nor the right atrium. The single stable spiral can be seen in Figure 5. The core of the spiral is denoted by an open circle in the leftmost panel of the RA-spiral and the rightmost panel in the LA-spiral on the top row of the voltage patterns.

For the second case, where the appendages are adipose remodeled, the observed dynamics is more complex. Both in the case of the right and the left atrium we obtain break-ups that disappear again over the course of time. The break-ups always occur in the right atrium. When the spiral is present in the right atrium, the breaks occur mostly around the atrial appendage and the *crista terminalis* (see the middle panel of the RA-spiral in Figure 5 in the second row of the voltage patterns). When the spiral is present in the left atrium, some breaks occur around the right atrial appendage (see the leftmost panel of the LA-spiral in Figure 5 in the second row of the voltage patterns). The reason breaks occur in the opposite atrium are due to the larger size of the right atrial appendage which has a high influence on its surroundings. In this last case of the spiral in the left atrium we can also see increased complexity of the wave pattern in the left atrium itself. Break-ups do occur, but are short-lived. A clear increase in complexity can be seen with respect to the previous case. The spiral in the left atrium also creates a more complex pattern than the one in the right atrium.

In the third case, with AF remodeled tissue together with AF and adipose remodeled appendages, even more complexity is observed. In both the cases of RA-spiral and LA-spiral additional transient spirals occur. In the RA-spiral case they appear mainly around the *crista terminalis* due to its fiber orientation, although some spirals may be observed around the right atrial appendage as well. In the case of the LA-spiral the disappearing spirals occur once again in the right atrium. There are however also new spirals created in the left atrium which do not disappear but instead remain active over the course of time. They occur due to the presence of the strongly pronounced fiber direction of Bachmann's bundle in combination with the waveblock that occurs due to the left atrial appendage. A total of five spirals is observed. Just as in the previous cases, all spiral cores are denoted by circles (permanent) or stars (transient) in Figure 5. It is again clear that the spiral in the left atrium makes for a more complex pattern than the one in the right atrium.

For all cases, movies can be found in the Supplementary Information.

## 4. Discussion

### 4.1. Findings

Adipose tissue is an important factor that contributes to arrhythmias. The fat is mainly located on the epicardial wall of the atria, or infiltrating in it. Epicardial adipose tissue infiltrates can cause local inflammation which results in fibrosis (Haemers et al., 2017). The arrhythmogenic consequences of fibro-fatty remodeling have been discussed earlier (De Coster et al., 2018). The possible effects for electrophysiological remodeling of neighboring myocytes by these adipocytes are not yet studied. Experimental data using patch clamp has revealed adipocyte induced remodeling of the cardiomyocyte electrophysiology (Lin et al., 2012). We use these results measured on rabbit atria, project them to human tissue and investigate the possible effects of the observed remodeling.

While only focusing on the ionic changes that happened to the cell, we were able to reproduce the correct cell-membrane voltage behavior. There is an increase in RMP, a higher plateau level and APD lengthening. Therefore, our model provides the same qualitative results as were observed experimentally.

In chronic AF, one typically observes shortening of APD duration (Nattel et al., 2008). The observed prolongation of the APD by adipocytes was therefore proposed as the possible mechanism for induction of the arrhythmia, by inducing further heterogenicity in APD within the atria. We observed in the model that due to ionic changes we have a more-positive resting membrane potential and a longer APD. These changes reduce the depolarizing threshold and result in easier induction of arrhythmia.

We show that in 2D monolayers complex spiral wave dynamics are seen. It is observed that heterogeneities cause arrhythmias, however, they terminate spontaneously. The minimal size of an adipose remodeled patch to create arrhythmia is substantially large. For smaller patches, the arrhythmia can be induced but it normally passes through and terminates. This would correspond to transient atrial flutter. Short episodes of arrhythmia occur as a result of high frequency pacing, however to be sustained the spiral should be able to rotate inside the adipose remodeled patch. This has to do with symmetry breaking. When a spiral is spontaneously induced inside a circular patch it will need more space since two symmetric spirals should be able to rotate. When the symmetry is broken, smaller sizes are needed to keep the arrhythmia alive. For spontaneously induced arrhythmia this means that one needs a large patch (larger than the atrial appendages in realistic human atria), while the S1S2 induced arrhythmias could be sustained in patches that are comparable to the size of a human atrial appendage.

We studied induced spirals in fully remodeled tissue as well and can see that they are persistent and create additional breaks. The arrhythmias are more complex under adipose remodeling than without it, both in the case of regular Courtemanche as AF Courtemanche. A single rotor seems to be necessary in realistic atria (smaller patches) to create AF. These rotors could be created, for example, due to structural remodeling of adipose tissue or fibrosis (De Coster et al., 2018). Once the spiral core is present inside the adipose remodeled patch, an increase in complexity of the spiral wave pattern, can be seen, corresponding to AF.

For realistic human atria, additional factors need to be taken into account. One of them is the geometry, another one is the fiber structure.

We see that arrhythmia complexity increases from regular Courtemanche to the adipose remodeled appendages model, and further for the AF and adipose remodeled appendages model. The arrhythmias were obtained by inducing a spiral with an S1S2-protocol. Spontaneously generating an arrhythmia was not successful. This can be attributed to the small size of the appendages.

The left atrium was clearly more prone to rotors than the right one due to the fact that the fiber orientation is inherently different in the left and right atrium. The current model has the fiber directions obtained from a mathematical model based on some measurements of anisotropy in few regions of atria (Dössel et al., 2012). Measuring of anisotropy of full atria would enhance the accuracy of simulation. Currently the left atrial appendage is not sufficiently large to obtain sustained arrhythmia. However, in AF it is known that the atria dilate which increases the size of the left atrial appendage. It would therefore be interesting to obtain anatomical data on dilated atrial appendages and study how it will affect the results on induction of the arrhythmia.

### 4.2. Limitations

Although the results show pronounced arrhythmogenic effects, we need to consider them with caution. Despite the adaptation of the model toward the human atrial myocyte, the original measurements of the ionic current remodeling were based on rabbit data. The experimental measurements were also performed with a short amount of time in co-culture (Lin et al., 2012). Chronic remodeling occurs over much longer time frames, which might result in different effects on the currents.

Rabit atrial cells don't behave exactly the same as human atrial cells however, despite the fact they show many similarities. The most notable difference is in the transient outward potassium current *I*_*to*_ (Wang et al., 1999). This current repolarizes slower for rabbit than for human. Since we looked at relative changes, it is assumed that no effects due to this different time-dependence will occur.

The temperature of the measured currents in Lin et al. (2012) was at room temperature. The temperature changes are present in both the regular myocytes as well as the adipose remodeled myocytes. We are always looking at relative changes in currents. Therefore, the parts that get affected by the temperature are assumed to cancel out once you take the relative value. For this reason, temperature dependence of the measured currents is not taken into account here. In addition, in Lin et al. (2012) only differences in the peak I-V values of major currents are measured. However, remodeling can affect not only the peak values, but also activation and inactivation kinetics of the currents. Changes in time kinetics of the channels may add additional effects on adipose remodeling and its effect on onset of arrhythmias. To account for these effects, more measurements of currents and their activation-inactivation kinetics in co-cultures with human atrial myocytes at body temperature should be done for different durations of contact with adipocytes. With that kind of data it will be possible to represent adipose remodeling not as voltage-dependent scaling factors but through a novel formulation of equations for each current separately. This can subsequently be used to study more accurately effects of adipose remodeling on arrhythmogenicity.

In our paper we used the standard Courtemanche model for normal and AF remodeled atrial tissue (Courtemanche et al., 1998, 1999). This model is one of the most widely used ones in atrial modeling (see e.g., Nattel et al., 2007; Pandit and Jalife, 2013). Recently new models for atrial remodeling were proposed. For example Loewe et al. (2014) proposed a model which accounts for chronic atrial fibrillation changes. It does so by changing potassium currents and increasing the cell capacitance based on a literature study. It will be interesting to find out if adipose tissue remodeling will have similar effects in this model and this should be addressed in a subsequent study. Possible dependency of the results of cardiac modeling on the cell model used, was also stressed in a recent study (Muszkiewicz et al., 2017), which investigated how variability in specific ionic currents impacts the phenotypic variability observed in human atrial cells. The authors compared three models: Courtemanche et al. (1998), Maleckar et al. (2009), and Grandi et al. (2011) and found that in order to reproduce the same experimental data, models need to be changed differently and thus the same changes in conductances of ionic currents in different model can produce different effects.

It should be noted that the readjustment factors of the currents derived from these measurements are only important for large values of the currents. When the current is close to zero, the form of the readjustment factor does not matter that much. Hence a plot that uses either a constant or a parabolic factor (e.g., *I*_*Na*_) is only important near the points where the current is non-zero (−80 mV < V < −20 mV). As in this range the readjustment factor is constant we chose the constant approximation. Even if outside of this range we can have substantial deviations from this approximation it will not affect the results. Another issue is the presence of an extra peak at 40 mV for ICaL in the I-V curve. This peak does not look physiological and occurs due to the combination of the behavior of the Courtemanche model together with our fit of the scaling factors. We decided to keep this representation and make no further adjustments for voltages V > 30 mV, as such voltage range is not reached in our simulations. However, it is obviously a limitation of this approach and should be possibly corrected in the future.

Earlier, chronic AF remodeling was already mentioned. It has been shown that adipose infiltrates are the beginning stage in the development toward chronic AF. We are therefore modeling the beginning stages of full scale chronic arrhytmia. The model that was currently used is the Courtemanche model and the AF model from Courtemanche. We apply adipose remodeling to both these models to look for possible effects on wave dynamics. However, it would be interesting to follow up these models and see how they evolve. More ionic changes as a result of adipose arrhythmogenicity could make the system end up in the cAF model from Loewe et al. (2014), or it might end up in an adipose remodeled version of that model. More research will need to be conducted to look at at the time evolution of the models such that they can end up in chronic atrial fibrillation.

In our 2D simulations with an adipose remodeled patch, the spiral wave was sustained only if it was rotating inside this patch. Such tendency of attachment of the spiral to the patch region can be understood as drift of the spiral wave to the regions with longer period. This was observed in various models of cardiac tissue (Rudenko and Panfilov, 1983; Ten Tusscher and Panfilov, 2003). In addition, such heterogeneities can potentially attract existing rotors, leading to anchoring Defauw et al. (2014) as it could be seen in the case of the smallest radius with the Courtemanche model and adipose remodeled patch.

In our simulations, spiral waves were initiated by an S1S2 protocol. It would also be interesting to study other mechanisms of initiation of spiral waves, such as due to curvature effects (Pertsov et al., 1983), or heterogeneity in the refractory period (Panfilov and Vasiev, 1991). One can then study if the mechanism of initiation will affect the dynamics of spiral waves. In addition, it would be interesting to study if ionic remodeling can affect the process of defibrillation of cardiac tissue (Keener and Panfilov, 1996).

We also did not study combined structural and ionic remodeling. This was done due to the size difference between our study of structural remodeling and the one of ionic remodeling. The three-dimensional model would not be able to distinguish between adipocyte obstacles and fibrotic obstacles. It would be interesting to do however when more detailed heart data will be available. A recent example of such data can be found in Stephenson et al. (2017). Such study will also be interesting because recent work by Morgan et al. (2016), showed that myocyte-fibroblast coupling in the atria also increases the resting membrane potential of the myocytes, similar to adipose remodeling. Thus, a combined consideration of fibrotic and adipose remodeling can further attenuate possible arrhythmogenic effects.

## 5. Conclusion

As an outlook toward further studies, we can say that the obstacle nature of an adipocyte creates conditions for the onset of arrhythmia, while the remodeling part makes it more persistent and increases complexity. Together they form the basis of an arrhythmia that is difficult to terminate.

This gives rise to two ways of handling adipose induced arrhythmias. On the one hand by focusing on prevention of adipose infiltrates themselves such that one can take and remove the trigger mechanism. On the other hand one could suppress the adipocytes from changing ionic properties which helps to make arrhythmias less complex.

We have shown that adipose tissue remodeling substantially affects the dynamics of existing cardiac arrhythmias leading to breakup and an increasing complexity of the activation pattern. However, onset of new arrhythmias requires that this remodeling occurs in substantially large regions. We also discussed the implications of the presence of adipose tissue and combined effects of AF and adipose remodeling.

## Author contributions

TDC and AP designed the numerical experiments. TDC wrote and tested the implementation of the model, performed the numerical experiments and did post-processing. GS provided the anatomical atrial voxel model. TDC, PC, and AP analyzed the results and prepared the draft of the manuscript. TDC, PC, GS, RW, KS, and AP critically revised the manuscript.

### Conflict of interest statement

The authors declare that the research was conducted in the absence of any commercial or financial relationships that could be construed as a potential conflict of interest.
