# Supplementary material for: Myocyte Remodeling Due to Fibro-Fatty Infiltrations Influences Arrhythmogenicity
Source: Front Physiol. 2018 Oct 4;9:1381. doi: 10.3389/fphys.2018.01381 (PMC6182296; doi:10.3389/fphys.2018.01381)
Supplement: Supplementary file 5 [file Data_Sheet_1.pdf]

Supporting Information

De Coster et al.

Video Legends

Video S1. ($SI\_2DSpiralDynamics.mp4$) S1S2 induced spirals in all four of the studied atrial myocyte models. The simulations display 10 seconds. Different activation patterns and spiral dynamics are observed for each cell model.

Video S2. ($SI\_PatchSpiral1.mp4$) Visualisation of the first case in Figure 4 of the main manuscript. It is seen with a Courtemanche configuration with an adipose remodelled patch of radius $1.536cm$. The spiral doesn't anchor immediately to the patch but needs some time. Eventually it does get there however.

Video S3. ($SI\_PatchSpiral3.mp4$) Visualisation of the third case in Figure 4 of the main manuscript: a spiral influencing remodelled patch. It is seen with a Courtemanche configuration with an adipose remodelled patch of radius $4.608cm$. Inside the patch some chaotic behaviour is present. Over the course of time, this chaotic behaviour produces waves which it pushes outside of the patch that interact with the spiral. This gives rise to behaviour that lies in between spiral anchoring and chaotic behaviour in the middle of the patch.

Video S4. ($SI\_3DSpirals.mp4$) Visualisation of Figure 5 in the main manuscript. Each row corresponds to the tissue types that were used (Courtemanche, Courtemanche + adipose remodelled appendages, AF Courtemanche + AF and adipose remodelled appendages). Each column corresponds to the location where the arrhythmia was initialised (in the right atrium, or in the left atrium). One can see different kinds of electrical wave propagation complexity for the different combinations present.

Supplementary Information Accompanied by Supporting Figures

Single Cell. In the main manuscript, there is made use of four different cell models. The four considered models are the Courtemanche model itself, the AF remodelled Courtemanche model, the adipose remodelled Courtemanche model and the AF + adipose remodelled Courtemanche model. All gating variables are described in the main manuscript. We provide them in Table S1 as well though in full with the aim of easy replication for future studies.

Table S1. Maximal conductances for selected currents in all four considered cell models.

gating variable (nS/pF)	**Courtemanche**	**Adipose remodelled Courtemanche**
$g(V)_{Na}$	7.8	8.814
$g(V)_{CaL}$	0.12375	$V < 0mV$: $0.000210375V^2 + 0.24193125$<br>$0 < V < 20$: $0.256 + (0.241 - 0.256)\left(1 + e^{0.4*(V-7.82mV)}\right)^{-1}$<br>$V > 20mV$: $0.00061875(V - 20mV)^2 + 0.2561625$
$g(V)_{to}$	0.1652	0.229628
$g(V)_{Kur}$	$0.005 + 0.05\left(1 + e^{-\frac{V-15mV}{13}}\right)^{-1}$	$0.0044 + 0.044\left(1 + e^{-\frac{V-15mV}{13}}\right)^{-1}$
$g(V)_{Ks}$	0.1294	$0.10352 + (0.05176 - 0.10352)\left(1 + e^{0.08*(V-30mV)}\right)^{-1}$
$g(V)_{K1}$	0.09	$0.05202 + (0.07245 - 0.05202)\left(1 + e^{0.3*(V+70mV)}\right)^{-1}$

gating variable (nS/pF)	**AF remodelled Courtemanche**	**AF + adipose remodelled Courtemanche**
$g(V)_{Na}$	7.8	8.814
$g(V)_{CaL}$	0.037125	$V < 0mV$: $0.0000631125V^2 + 0.072579375$<br>$0 < V < 20$: $0.0768 + (0.0723 - 0.0768)\left(1 + e^{0.4*(V-7.82mV)}\right)^{-1}$<br>$V > 20mV$: $0.000185625(V - 20mV)^2 + 0.07684875$
$g(V)_{to}$	0.0826	0.114814
$g(V)_{Kur}$	$0.0025 + 0.025\left(1 + e^{-\frac{V-15mV}{13}}\right)^{-1}$	$0.0022 + 0.022\left(1 + e^{-\frac{V-15mV}{13}}\right)^{-1}$
$g(V)_{Ks}$	0.1294	$0.10352 + (0.05176 - 0.10352)\left(1 + e^{0.08*(V-30mV)}\right)^{-1}$
$g(V)_{K1}$	0.09	$0.05202 + (0.07245 - 0.05202)\left(1 + e^{0.3*(V+70mV)}\right)^{-1}$

The gating variables rescaling factors were based upon simulations of patch clamp protocols to reproduce I-V curves. To give validation for the correct use of them in the main manuscript, here it is shown that the exact results from the paper of *Courtemanche et. al.* (1) can be reproduced. In Figure S1, the process of constructing an IV curve is demonstrated. One cell was put on a holding voltage of $-80mV$ for $10s$. After this was done, the holding voltage was changed and set to a fixed value. The currents were then measured for a period of $3s$ under this constant voltage.

For the current I_{CaL}, this process is visualised in sub-panel A and B for a variety of holding potentials, going from $-50mV$ up to $50mV$. Sub-panel A shows the current for a duration of $1.5s$ after the onset of the new holding potential. Sub-panel B shows a zoomed in version of the sub-panel A where all the peaks can be seen.

To arrive at an I-V curve, the maximum of each patch-clamp simulation should be taken. When these maximal currents are then plotted against voltage, an I-V curve is realised. This has been done for all currents. The ones plotted in sub-panel C are I_{Na}, I_{Kr}, I_{CaL}, I_{Kur}, I_{to} and I_{Ks}. This figure can be compared to Figure 3 in the paper of *Courtemanche et. al.* (1), which shows a perfect match between the two.

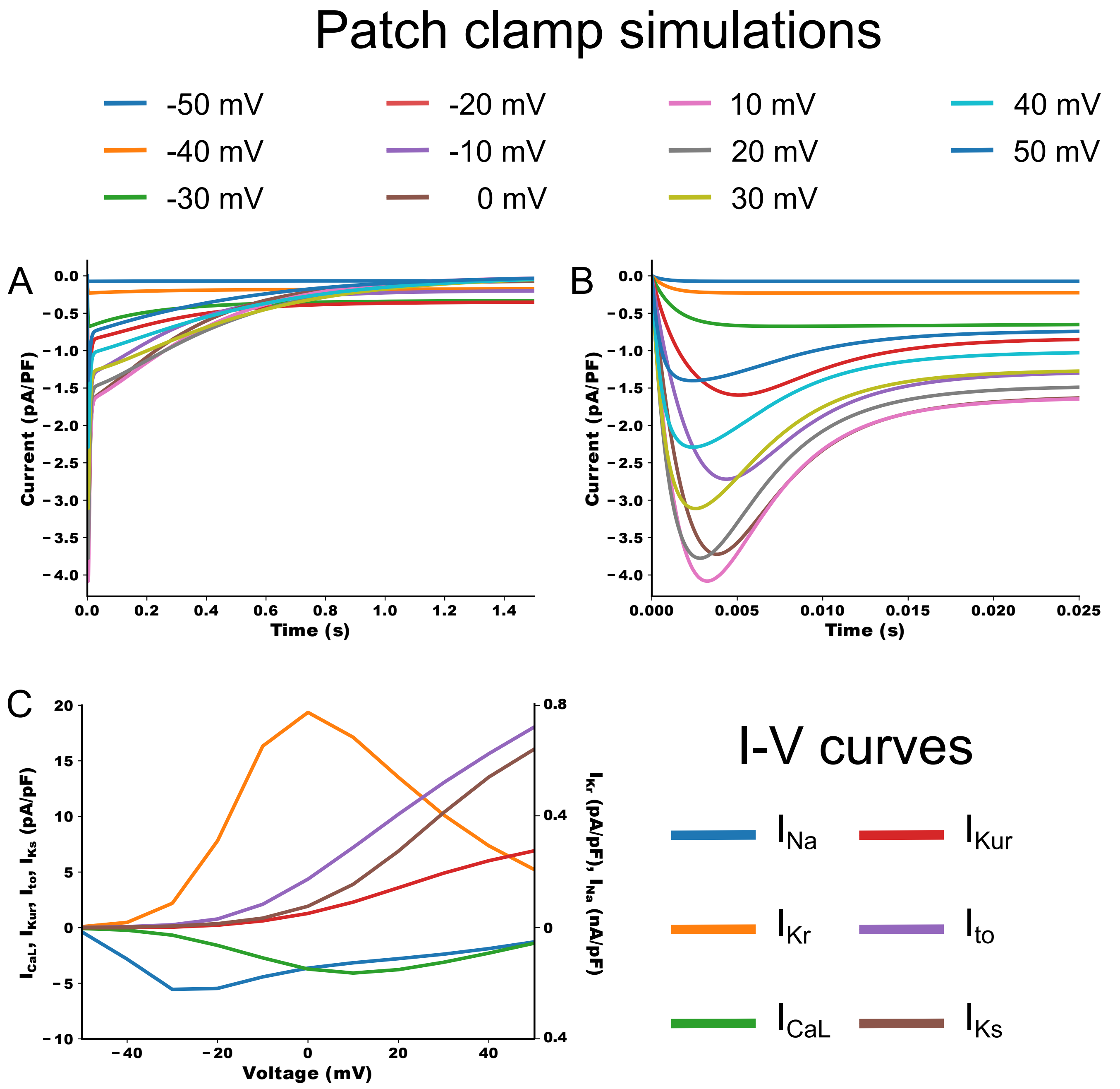

Fig. S1. The process to construct an I-V curve is visualised here. Panel A and B show in silico patch clamp experiments, while panel C shows the eventual result for a variety of currents.

Adipose remodelling is a result of a change of six ionic currents. We studied the contributions of each of these currents on the AP shape and duration separately. The results are shown in Figure S2. Here Figure S2A shows the AP shape when only a single current was changed, Figure S2B shows the difference in shape with the Courtemanche AP, and Figure S2C explores the linearity of the contributions. When looking at these separate contributions, it can be seen that the I_{Na} increase makes for the initial higher upstroke (it just coincides with the orange line at the upstroke). The increase in I_{CaL} together with the decrease in I_{Kur} rise the plateau level. The prolongation of this plateau level and the long phase III in the AP are mediated through the changes in the three remaining channels, namely an increase in I_{to}, a decrease in I_{Ks} and a decrease in I_{K1}. However, these separate changes alone do not predict the overall changes. In Figure S2C we see that if we add the individual contributions (the blue line) we obtain more substantial changes in AP shape than when they are modified together (the orange line). This means that some of these currents counteract each other in a complex non-linear way.

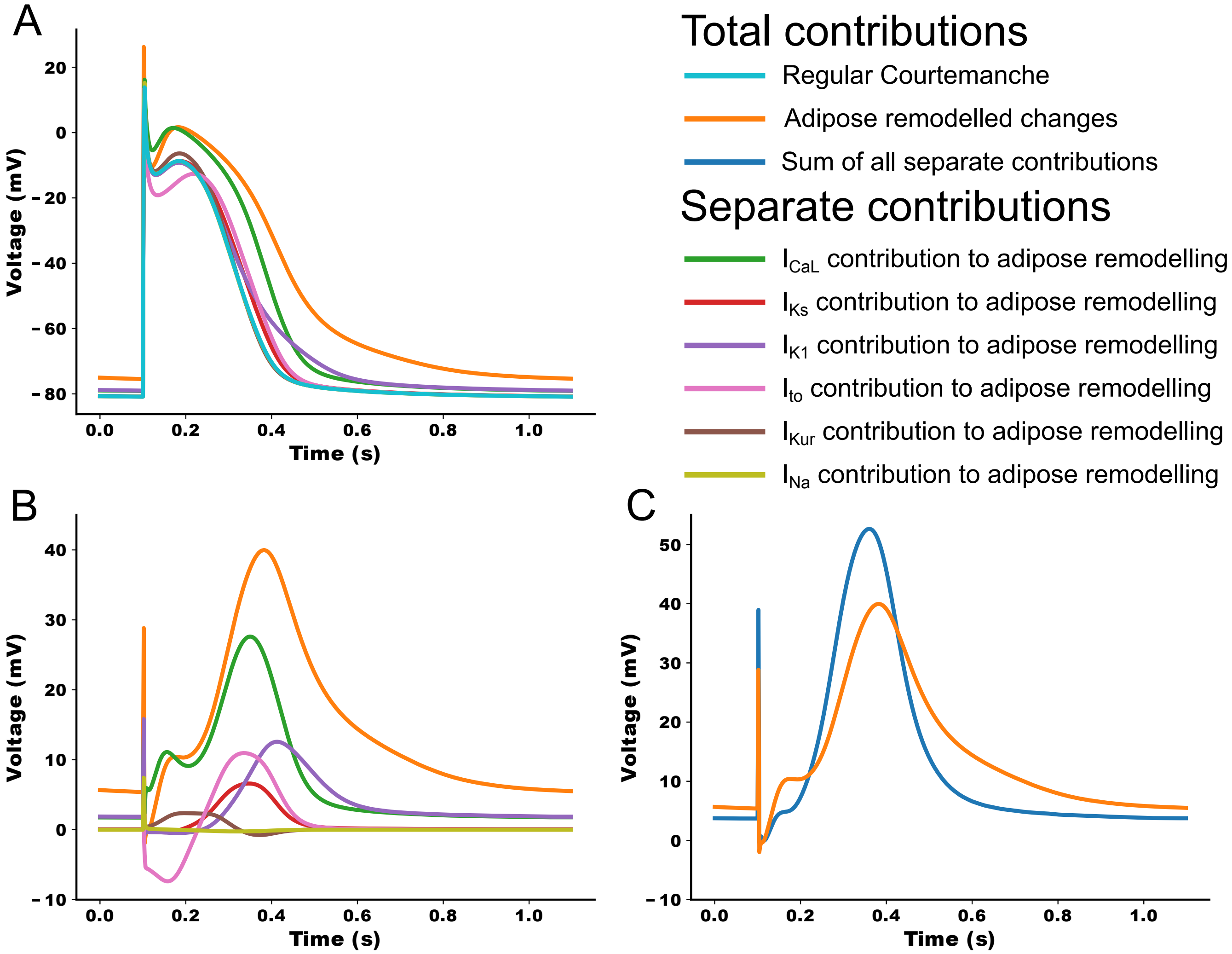

Fig. S2. The changes in ionic currents due to the presence of adipose tissue and corresponding action potentials (A). All individual contributions of each of the six adapted currents is shown as well. In the bottom left graph (B), the differences between the regular Courtemanche model and the adapted models is shown. The bottom right graph (C) shows the difference of the adipose remodelled model with the Courtemanche model, as well as the sum of all the differences of the individual currents.

1D Propagation.

Restitution curve. In addition to the restitution curve we further characterize rate dependency in the following way.

In Figure S3 we plotted the relative APD-change as a function of pacing frequency. For each frequency, the APD_{90} was divided by the corresponding APD_{90} of the Courtemanche model. In this way, the relative behaviour of the models can be seen. The blue, orange and green lines stop at the maximal possible frequency value for the corresponding model which is $1.4, 2.1$ and $4.9Hz$. We see that the red line approaches the green line at higher frequency, which shows that the degree of AF remodelling decreases with increase in frequency. Adipose remodelling, on the contrary, slightly increases with frequency increase.

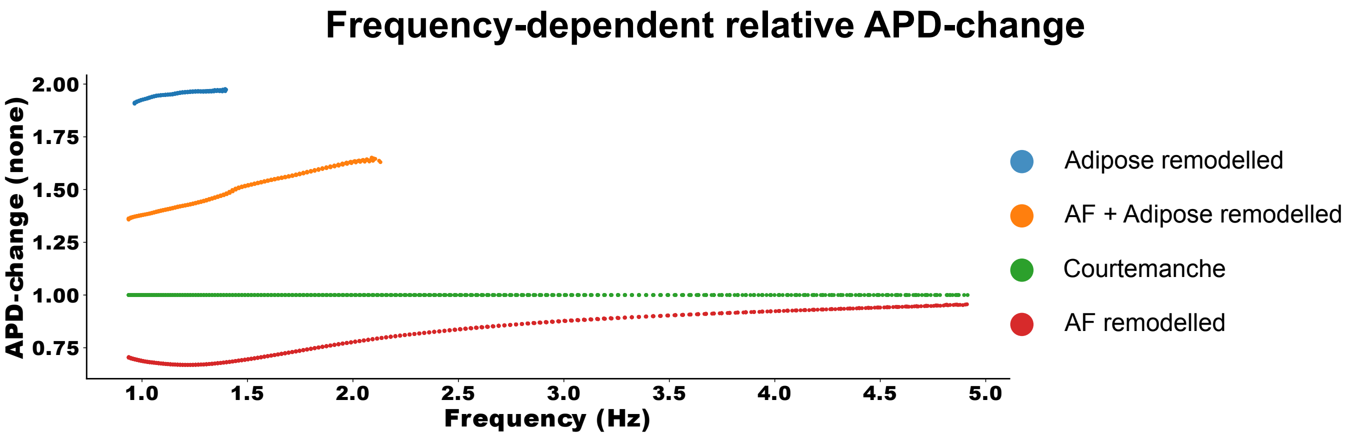

Fig. S3. The relative changes in APD_{90} with respect to the frequency of pacing. The curve was made for all four models relative to the Courtemanche model (green constant curve at value 1). More information on how this curve originated and the significance can be found in the text.

References

1. Marc Courtemanche, Rafael J Ramirez, and Stanley Nattel. Ionic mechanisms underlying human atrial action potential properties: insights from a mathematical model. *American Journal of Physiology-Heart and Circulatory Physiology*, 275(1):H301–H321, 1998.
